# The potential role of illness expectations in the progression of medical diseases

**DOI:** 10.1186/s40359-019-0346-4

**Published:** 2019-11-08

**Authors:** Francesco Pagnini

**Affiliations:** 1 Department of Psychology, Università Cattolica del Sacro Cuore, Via Nirone, 15, 20123 Milan, Italy; 2 Department of Psychology, Harvard University, Cambridge, MA USA

**Keywords:** Illness expectation, Chronic disease, Mind-body connection, Health Psychology, Placebo, Nocebo

## Abstract

To what extent can one’s mind promote direct changes to the body? Can one’s beliefs about the body become a physical reality, without mediating effects from behaviors? Specifically, can medical symptoms and the course of a disease be directly affected by a person’s mindset about the illness?

There is a vast literature about placebo and nocebo effects, that promote physical changes by creating the expectation of a change through a primer (for example, a fake pill). Placebos, however, often imply deception, or at least ambiguity, to be effective. The concept of Illness Expectation describes the expectations, both implicit and explicit, that a person who has received a diagnosis makes about the course of the disease. It can be characterized by different degrees of rigidity, and it is argued here that these expectations can ultimately lead to changes in the disease progression. These changes may happen through behavior modifications, or through a non-behavioral pathway, which may deserve exploration efforts from the scientific literature.

## Mind, body, and placebo

The relationship between mind and body has a long history in both medicine and psychology. Beginning with Descartes, the mind/body concepts were based on a strict dualism. More recent investigations, however, demonstrate a strong interaction between mind and body, effectively collapsing the dualistic construct [1]. Among the most studied effect that the mind exerts on the body is the placebo/nocebo effect, in which physiological changes emerge following the assumption of inert or non-specific treatment components [2]. As there is no active therapeutic component in placebos, their effects are generally attributed to the patient’s beliefs of efficacy of the treatment. Placebo effects, or placebo response, refer to the desirable effects, either subjective (psychological) or objective (physiological), while nocebo effects refer to the anticipated negative effects promoted by a treatment (e.g., side effects). Given their clinical relevance, there has been a growing interest in studying the placebo and nocebo mechanisms, though there are still several open questions [3]. For example, deception (e.g., not informing the patient that the pill is inert) or, more commonly, ambiguity (e.g., patients are told that it is uncertain what treatment they are receiving) can be a source of ethical challenges. Of course, the placebo response is not always obtained through a lack of, or limited information (see, for example, the emerging open-label placebo model [4, 5]). Moreover, both placebo and nocebo responses are not restricted to inert or “fake” interventions, but they can modulate the effects of drugs and other therapies [6]. The placebo effect depends on several aspects [7], including psychological processes (e.g., implicit learning and previous experiences), social and contextual factors (e.g., the patient-provider relationship, treatment characteristics), and biological mechanisms (body’s healing properties and neurophysiological processes). The beliefs about the intervention are also influenced by psychological traits, such as optimism [8] and spirituality [9], even though situational variables seem to play a bigger role for the placebo effects than individual characteristics [10].

## Effects of expectations on the body

One of the main operational mechanisms of placebos is represented by cognitive expectations, which in turn are expected to promote the occurrence of physiological changes in the body [11]. In general, placebo and nocebo effects have been studied with a primer, such as a sugar pill, that influences or conditions the person to anticipate an effect. The expectation of a medical effect promotes both subjective and objective (physiologic) changes, with clinical improvements or worsening [12]. However, expectations are not only prompted by drugs or interventions. In fact, every individual with a medical condition develops a certain mindset toward the illness [13], with expectations that spontaneously emerge. These expectations, which represent the result of the elaboration process of the information collected about the disease [14], can promote different physiological effects [15]. For example, blood glucose levels in people with type II diabetes are influence by perceived time and expected values, rather than being a mere physiological process [16]. Furthermore, expectations can influence the ageing process: older adults who think about ageing as associated with negative characteristics tend to experience a greater loss of physical function and a reduced survival, compared to those who held positive expectations [17].

## Illness perceptions and health beliefs

Expectations about the disease are a central component of illness perceptions and health beliefs, which are well-established concepts in health psychology [18]. Illness Perception is often explored within the theoretical framework of the Common Sense Model (CSM) of Illness Representation [19]. In the CSM theory, patient’s illness perceptions include beliefs about what precipitated the illness (causes), how long it will last (timeline), the impact on the patient’s life (consequences), which symptoms are attributed to the illness (identity), and how the condition can be controlled or cured by the patient’s behavior (personal control) or by the treatment (treatment control). In the CSM, expectations are considered as an underlying component of the different beliefs [20, 21]. Emotional components are another key aspect of the CSM, which may interfere with cognitive processing, and it could be a source of confusion during the assessment process. For example, one of the most utilized instruments for the assessment of illness perception, the Brief Illness Perception Questionnaire [22] includes items like “How much does your illness affect you emotionally?”, which are somehow related to the expectations, but refer directly to the emotional domain. The same concern deals with questions about consequences in everyday life (e.g., “My illness has serious economic and financial consequences”, from the Illness Perception Questionnaire Revised [23]).

Thus far, most published research referencing the Illness Perception construct focuses on the role of disease representations in explaining both coping and outcomes in patients with a wide range of health conditions [24, 25]. Specifically, health psychologists have explored how disease representations can lead to lifestyle modifications, eventually leading to changes in the medical outcomes [26]. For example, adherence to the medical treatment, or lifestyle choices like eating, exercising, or smoking, can be influenced by illness representations. A person who perceives that nothing can change the course of the disease, for example, may be more prone to avoid exercising or taking prescribed medicine [27]. In other words, the effects of Illness Perceptions on the body (namely, on the course of the disease or its symptoms) have been mainly explored as mediated by behavior changes [28]. The main difference between the construct of Illness Perception and Illness Expectation is their specificity: while the former is a multifaceted concept that includes several aspects of the illness experience, the latter is a specific element, the anticipation of the future illness-related scenarios, which is merely cognitive.

## Emotions and somatic changes

While the influence of psychological factors on the body has been explored with the mediating effect of behavior changes, there is also a vast literature that has investigated the relationship between negative affects, such as stress and depression, and medical outcome. Fields such as psychoneuroendocrinology and psychoneuroimmunology have been explicitly created to investigate these relationships. Briefly, we know that negative emotions (e.g., depression, stress) have, among other effects, a strong impact on human physiology [29], often reflecting on poorer medical outcomes, in the case of chronic diseases. For example, depressive states and stress have been associated with reduced survival rate in patients with cancer [30]. The mechanisms underlying these associations are still under investigation.

## Illness expectations

Despite the vast placebo/nocebo literature, expectations are not typically manipulated directly and are often discussed in unison with an inert agent. Expectations seem to have a role in illness beyond the delivery of (fake) treatments. This situation leaves some ambiguity when the findings on placebo effects apply and when they do not. For this reason, it could be relevant to isolate a specific model, that specifically refers to the expectations that a person has toward his/her illness. I suggest to define this construct “Illness Expectation” (IE). IE is the cognitive schema that defines the expected characteristics of the disease progression and future-oriented beliefs about the symptoms. Like other expectations [20], IEs can manifest as explicit (conscious) future-directed cognitions, or they may be implicit, without an individual’s full awareness. There could be a certain level of overlap between conscious and unconscious processes [31], but though they do not necessarily converge. For example, a person can be well-informed about the expected trajectory of his/her disease, but it is also possible that, implicitly, (s)he represents future developments of the symptoms in a different way. The expectations are based on the supposed knowledge about the diagnosis and the illness [32]. They are therefore influenced by the information received, as well as by the cognitive and emotional elaboration processes, which rely on personal history and skills. Verbal information, patient-clinician interactions, and prior experiences or previous conditioning, as well as personality and other psychological factors (e.g., optimism) may influence the expectation creation, similar to how they influence the placebo response [33, 34]. Illness Expectations could be seen as a specific form of response expectancy, defined by Kirsch [35] as the anticipation of non-volitional responses.

As with other psychological constructs, IEs reflect individual differences, that is, people with the same diagnosis and who have received similar information may have different expectations. The same psychological traits that influence the development of the treatment expectations, which may module the placebo response, may be involved in the characterization of Illness Expectations. For example, it is possible that optimism and spirituality have a positive impact, which would be in line with the positive associations found with these two variables and health [36]. At the same time, social and contextual aspects, such as the patient-physician relationship and trust, social support, could play a role in modulating the expectations. Future studies are required to understand the possible role of these variables.

A crucial factor that mediates the effects of IE is cognitive rigidity. As a cognitive schema, expectations may additionally incorporate different degrees of rigidity, ranging from a mild expectation to a very strict conception of what “will” happen in terms of disease progression. The concept of rigidity, in this context, refers to an inability to maintain a dynamic view of one’s status, effectively keeping evaluations static over time [37]. In other words, rigid IE tend to be very emphatic and resistant mental sets, which could be similar to certain core beliefs in the cognitive-behavioral approach [38]. It is effectively a form of mindlessness, in which an idea is unchanged over time even with changes in situation or context [39, 40]. Cognitive rigidity, which is the reverse of cognitive flexibility, is generally considered a stable characteristic over time [41]. Similar to flexibility, however, rigidity could change over time, for example as a result of a psychological intervention [42].

Under the lens of the Illness Expectation model, the placebo response is not necessarily the arising of new, treatment-related expectations, but it could represent the modification of a previously existing mindset. However, the IE effects are not limited to placebo responses. Placebos are an external manipulation, often achieved with some form of ambiguity or deception (e.g., a “fake” pill), while IEs are self-created, although they can be influenced by external manipulations (e.g., doctor’s opinions, information from other patients).

**Fig. 1:**
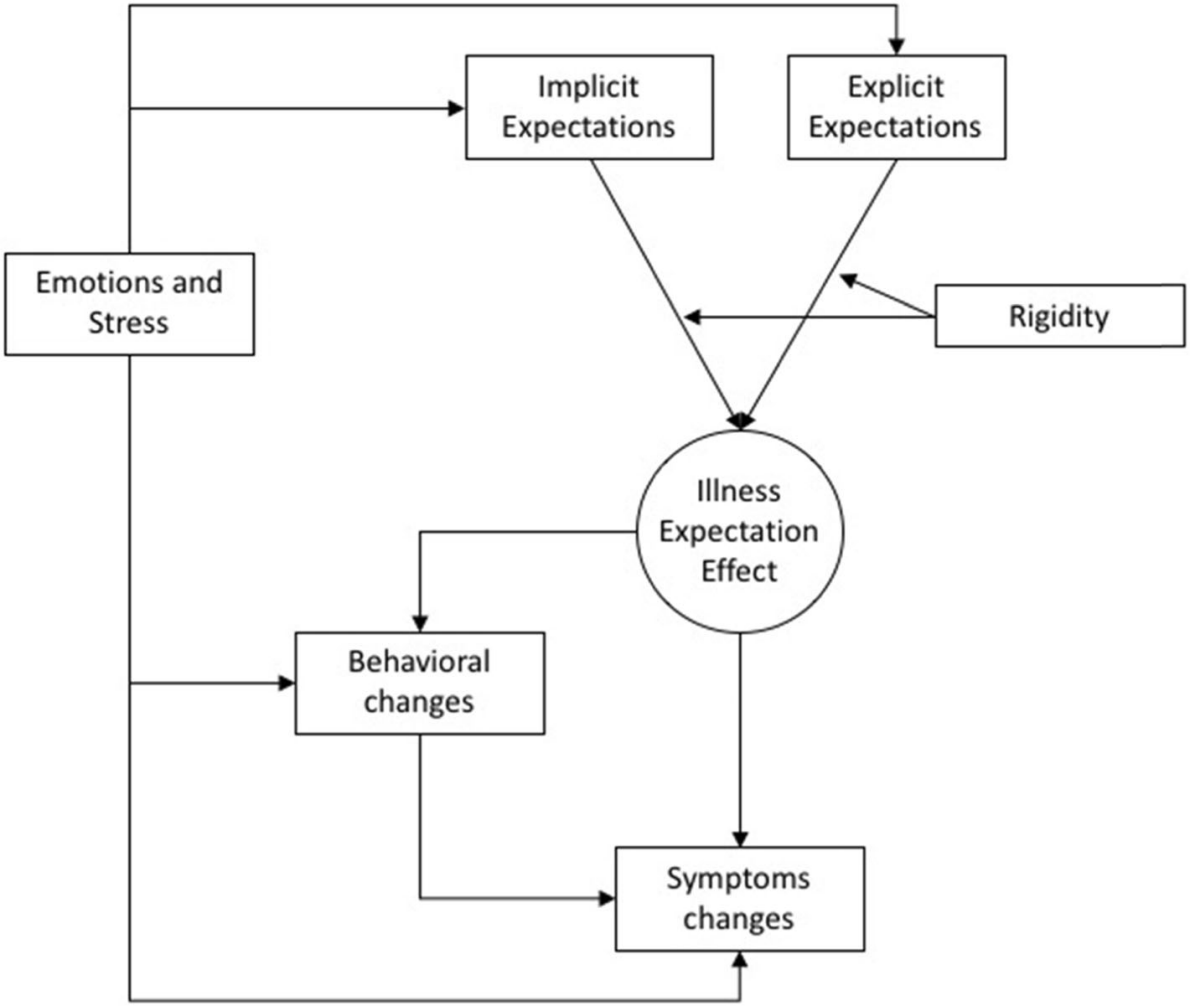
Illness Expectation model

It is here suggested that IE could influence symptoms and disease progressions (i.e., medical outcomes) with two ways: a behavioral way and a non-behavioral way (Fig. 1). The former refers to behavioral changes, including adherence to the treatment and lifestyle (physical activity, eating habits…) modifications. The non-behavioral way refers to the physiological changes “directly” influenced by the expectations, mirroring the placebo/nocebo effect, but observed without a primer.

While expectations and rigidity focus emphasize the cognitive level of the mind/body interaction, emotions and stress can also interact with the process, with different pathways. Emotions (e.g., fear) could influence both implicit and explicit expectations. They could lead to behavioral changes, and direct effects of negative emotions on the body (e.g., immune system) are documented [29]. A peculiarity of this model is the role of rigidity, which could represent a clinical target for psychological interventions. There are several psychological approaches that could improve flexibility and discourage rigid thinking. Future studies could explore how these interventions could modify the IE effects on the body.

The IE model, at the present, is based on indirect evidence from the scientific literature and organized through this theory. Empirical studies are warranted to test its validity and provide direct data-driven conclusions. One of the first problems that should be addressed by this field is the development of tools for the expectation assessment. While there are existing instruments that assess expectations, most of them focus on treatment expectations. For example, The Credibility/Expectancy Questionnaire [43] explores treatment credibility and expectancy, while the Stanford Expectations of Treatment Scale [44] considers both positive and negative expectations. Although very important, treatment expectancies do not inglobe the illness expectations as a whole. Furthermore, considering the potential role of both implicit and explicit components, self-reported measures may not be able to fully assess the construct. The use of instruments to assess implicit components should be considered. Studying the effects of IE manipulations on the body may provide important confirms/disconfirms to the mind/body connection hypothesis, with the potential to lead to several clinical implications. Perhaps the most important and ambitious one would be a better understanding of how we can use a mechanism similar to the placebo effect, without the ethical burden of deception. The meaningful use of the placebo effect without deception has been suggested as a highly relevant research topic in psychology [3]. It could push to the limits our current understanding of the mind/body connection, with yet to be explored opportunities for clinical interventions.

## Data Availability

Not applicable.

## Abbreviations

CSM: Common Sense Model
IE: Illness Expectation

## References

[1] Phillips D, Pagnini F. Health and the psychology of possibility. In: *Critical Mindfulness* edn: Springer. 2016:173–82.

[2] Benedetti F (2014). Placebo effects: from the neurobiological paradigm to translational implications. Neuron.

[3] Geers AL, Miller FG (2014). Understanding and translating the knowledge about placebo effects: the contribution of psychology. Current opinion in psychiatry.

[4] Charlesworth JE, Petkovic G, Kelley JM, Hunter M, Onakpoya I, Roberts N, Miller FG, Howick J (2017). Effects of placebos without deception compared with no treatment: a systematic review and meta-analysis. Journal of Evidence-Based Medicine.

[5] Kaptchuk TJ, Friedlander E, Kelley JM, Sanchez MN, Kokkotou E, Singer JP, Kowalczykowski M, Miller FG, Kirsch I, Lembo AJ (2010). Placebos without deception: a randomized controlled trial in irritable bowel syndrome. PLoS One.

[6] Kleine-Borgmann J, Bingel U: Nocebo effects: neurobiological mechanisms and strategies for prevention and optimizing treatment. In: *International review of neurobiology. Volume 138*, edn. Edited by Colloca L: Elsevier; 2018: 271–283. 10.1016/bs.irn.2018.02.005 29681330

[7] Zion SR, Crum AJ: Mindsets matter: a new framework for harnessing the placebo effect in modern medicine. In: *International review of neurobiology. Volume 138*, edn.: Elsevier; 2018: 137–160. 10.1016/bs.irn.2018.02.002 29681322

[8] Geers AL, Wellman JA, Fowler SL, Helfer SG, France CR (2010). Dispositional optimism predicts placebo analgesia. J Pain.

[9] Hyland ME, Geraghty AW, Joy OE, Turner SI (2006). Spirituality predicts outcome independently of expectancy following flower essence self-treatment. J Psychosom Res.

[10] Meissner K: Believing in the effectiveness of treatment: from placebo to credition and back. In: *Processes of Believing: The Acquisition, Maintenance, and Change in Creditions.* edn. Edited by Angel HF, Oviedo L, Paloutzian RF, Runehov A, Seitz RJ: Springer; 2017: 125–137.

[11] Finniss DG, Kaptchuk TJ, Miller F, Benedetti F (2010). Biological, clinical, and ethical advances of placebo effects. Lancet.

[12] Benedetti F, Carlino E, Piedimonte A (2016). Increasing uncertainty in CNS clinical trials: the role of placebo, nocebo, and Hawthorne effects. The Lancet Neurology.

[13] Crum AJ, Zuckerman B (2017). Changing mindsets to enhance treatment effectiveness. Jama.

[14] Blasini Maxie, Corsi Nicole, Klinger Regine, Colloca Luana (2017). Nocebo and pain. PAIN Reports.

[15] Crum AJ, Leibowitz KA, Verghese A (2017). Making mindset matter. Bmj.

[16] Park C, Pagnini F, Reece A, Phillips D, Langer E. Blood sugar level follows perceived time rather than actual time in people with type 2 diabetes. Proc Natl Acad Sci. 2016;201603444. 10.1073/pnas.1603444113 PMC4961154 27382161

[17] Dionigi RA. Stereotypes of aging: their effects on the health of older adults. Journal of Geriatrics. 2015;2015.

[18] Petrie K, Weinman J (2006). Why illness perceptions matter. Clinical Medicine.

[19] Leventhal H, Meyer D, Nerenz D (1980). The common sense representation of illness danger. Contributions to medical psychology.

[20] Laferton JA, Kube T, Salzmann S, Auer CJ, Shedden-Mora MC (2017). Patients' expectations regarding medical treatment: a critical review of concepts and their assessment. Front Psychol.

[21] Cameron LD, Leventhal H: The self-regulation of health and illness behaviour: psychology press; 2003.

[22] Broadbent E, Petrie K, Main J, Weinman J (2006). The brief illness perception questionnaire. J Psychosom Res.

[23] Moss-Morris R, Weinman J, Petrie K, Horne R, Cameron L, Buick D (2002). The revised illness perception questionnaire (IPQ-R). Psychol Health.

[24] Hagger MS, Orbell S (2003). A meta-analytic review of the common-sense model of illness representations. Psychol Health.

[25] Petrie K, Weinman J (2012). Patients’ perceptions of their illness: the dynamo of volition in health care. Curr Dir Psychol Sci.

[26] Sarafino EP, Smith TW: Health psychology: biopsychosocial interactions: John Wiley & Sons; 2014.

[27] Upton D, Thirlaway K (2014). Promoting healthy behaviour: a practical guide.

[28] Petrie K, Jago LA, Devcich DA (2007). The role of illness perceptions in patients with medical conditions. Current opinion in psychiatry.

[29] Kiecolt-Glaser JK, McGuire L, Robles TF, Glaser R (2002). Emotions, morbidity, and mortality: new perspectives from psychoneuroimmunology. Annu Rev Psychol.

[30] Satin JR, Linden W, Phillips MJ (2009). Depression as a predictor of disease progression and mortality in cancer patients. Cancer.

[31] Colloca L, Miller FG (2011). How placebo responses are formed: a learning perspective. Philosophical Transactions of the Royal Society B: Biological Sciences.

[32] Rief W, Petrie KJ (2016). Can psychological expectation models be adapted for placebo research?. Front Psychol.

[33] Corsi N, Colloca L (2017). Placebo and nocebo effects: the advantage of measuring expectations and psychological factors. Front Psychol.

[34] Petrie KJ, Rief W (2019). Psychobiological mechanisms of placebo and nocebo effects: pathways to improve treatments and reduce side effects. Annu Rev Psychol.

[35] Kirsch I (1985). Response expectancy as a determinant of experience and behavior. Am Psychol.

[36] Koenig HG (2015). Religion, spirituality, and health: a review and update. Advances in mind-body medicine.

[37] Schultz PW, Searleman A: Rigidity of thought and behavior: 100 years of research. Genetic, social, and general psychology monographs 2002, 128(2):165. 12194421

[38] Dobson KS (2013). The science of CBT: toward a metacognitive model of change?. Behav Ther.

[39] Langer E: Mindfulness: Addison-Wesley/Addison Wesley Longman; 1989.

[40] Pagnini F, Philips D (2015). Being mindful about mindfulness. Lancet Psychiatry.

[41] Armbruster DJ, Ueltzhöffer K, Basten U, Fiebach CJ (2012). Prefrontal cortical mechanisms underlying individual differences in cognitive flexibility and stability. J Cogn Neurosci.

[42] Scott W, Hann KE, McCracken LM (2016). A comprehensive examination of changes in psychological flexibility following acceptance and commitment therapy for chronic pain. J Contemp Psychother.

[43] Devilly GJ, Borkovec TD (2000). Psychometric properties of the credibility/expectancy questionnaire. J Behav Ther Exp Psychiatry.

[44] Younger J, Gandhi V, Hubbard E, Mackey S (2012). Development of the Stanford expectations of treatment scale (SETS): a tool for measuring patient outcome expectancy in clinical trials. Clinical Trials.

